# Children’s Vantage Point of Recalling Traumatic Events

**DOI:** 10.1371/journal.pone.0162030

**Published:** 2016-09-20

**Authors:** Katie S. Dawson, Richard A. Bryant

**Affiliations:** School of Psychology, University of New South Wales, Sydney, Australia; Harvard Medical School, UNITED STATES

## Abstract

This study investigated the recollections of child survivors of the 2004 Asian tsunami in terms of their vantage point and posttraumatic stress disorder (PTSD) responses. Five years after the tsunami, 110 children (aged 7–13 years) living in Aceh, Indonesia were assessed for source of memories of the tsunami (personal memory or second-hand source), vantage point of the memory, and were administered the Children’s Revised Impact of Event Scale-13. Fifty-three children (48%) met criteria for PTSD. Two-thirds of children reported direct memories of the tsunami and one-third reported having memories based on reports from other people. More children (97%) who reported an indirect memory of the tsunami recalled the event from an onlooker’s perspective to some extent than those who recalled the event directly (63%). Boys were more likely to rely on stories from others to reconstruct their memory of the tsunami, and to adopt an observer perspective. Boys who adopted an observer’s perspective had less severe PTSD than those who adopted a field perspective. These findings suggest that, at least in the case of boys, an observer perspectives of trauma can be associated with levels of PTSD.

## Introduction

How people remember traumatic events is a critical factor in the adaptation to a trauma [1]. Memory for trauma in children has not been studied as comprehensively as it has in adults. This is a significant gap in the literature because children tend to have higher rates of posttraumatic stress disorder (PTSD) than adults [2]. Depending on the severity of trauma exposure, as many as one-third of children may develop PTSD [3], with one study reporting as many as 41% of tsunami-affected children having PTSD [4]. The prevalence of PTSD in children warrants further examination of the processes involved in its development, such as those relating to role of memory. Moreover, childhood memory for trauma is rarely studied in non-western settings. Accordingly, the goal of this study was to investigate the memories of child survivors of a major natural disaster in a non-western setting.

Understanding the role of memory in children’s stress reactions to trauma is important because of the central role of memory in perpetuating the stress response. Impairments in memory is a cardinal feature of PTSD, producing re-experiencing symptoms such as nightmares, intrusive memories and repetitive trauma play in children specifically [5]. The presence of these symptoms is seen to be indicative of a poor elaboration and processing of the trauma memory [6]. Much research has shown that analogous to adults, children with PTSD can suffer enduring re-experiencing memories [5] and that traumatic events, such as natural disasters, can have profound effects on children’s psychosocial development (for review see, [7]).

While the durability of childhood trauma memories has been contested in the literature [8], studies have consistently demonstrated the preservation of some details of traumatic events that occur in childhood. For instance, a 17-year follow-up study of survivors of a disaster found that even the youngest survivors (two-years old at the time) retained some memory of the event [9]. All of the children (three to four-year-olds) interviewed following Hurricane Andrew recalled the event [10]. Additionally, a series of studies conducted by Howe [11, 12] exploring the enduring nature of children’s memories for painful and invasive medical procedures found that despite a substantial decline a year later in recall of “peripheral” aspects of the event (e.g. who took the child home after the procedure), children could accurately recall central features of the event (e.g. details of the procedure).

Despite these similarities with adult memory for trauma, there are key differences in memory across the developmental trajectory (for a review, see [13]. Although very young children can demonstrate memory of previously seen events as young as nine months of age and by 18 months, they can recall complex sequences of novel experiences [14], long-term memory storage only occurs at a later stage in development. Young children can access memories when they are as young as two or three years of age [15], but these memories become inaccessible as the child ages, resulting in the well-documented pattern of childhood amnesia of events prior to three years of age [16].

As verbal skills develop, children begin to understand and interact with those around them, and they develop greater capacity to understand and contextualize their past in the form of autobiographical memories [17]. During this process, they rely on parents and others to assist in talking about the past, which is reflected in much evidence of the impact of parental reminiscing on children’s autobiographical memories [18, 19]. Consistent with this evidence, most theories recognise that a key difference in how children recall their experiences is shaped markedly by the extent to which their caregivers (usually their mothers) express reminiscing styles [20]. Typifying this perspective is Nelson and Fivush’s social-cultural theory, which posits that the social interactions in which a child develops shapes their self-construct and accordingly determines the nature and structure of memories of their past [21].

One clear implication from the social-cultural model is the influence of cultural context on autobiographical memories. Many studies have shown differential patterns of autobiographical memory in western and non-western samples across adult and child populations. For instance, several studies have demonstrated that western populations tend to retrieve earlier [22, 23], more collective [24], and more detailed [25, 26] memories than non-western counterparts. Although much research has been conducted cross-culturally in relation to children’s general autobiographical, there is relatively less work done on children’s memory for trauma across cultural contexts. This study seeks to explore features of traumatic memories in children from a south-east Asian background.

Relevant to the issue of trauma memory and one of the features primarily explored in this study is the vantage point from which autobiographical memories are recalled. Researchers have long noted that autobiographical memories can be recalled from one’s own perspective (“field”) or from a distant perspective (“observer”), such as seeing the experience from another’s visual perspective rather than through one’s own eyes. Commentators have argued that memories recalled from an observer perspective may function as a defense mechanism to avert unwanted emotional states [26]. This view is supported by numerous studies that observer perspective memories are less emotionally intense than those taking a field perspective [26–28].

In the context of clinical disorders, emerging research has demonstrated that adopting an observer perspective when recalling a traumatic event represents a form of cognitive avoidance that regulates emotional arousal, and may preclude emotional processing of the event [29–31]. Cognitive models of PTSD posit that avoidance, including avoiding memories of a traumatic event, plays a pivotal role in the development and maintenance of symptoms of the disorder [32, 33]. One prospective study found that adopting an observer perspective shortly after a motor vehicle accident was significantly associated with PTSD symptom severity during their hospitalisation and 12 months later [30]. However, to date this literature has given greater attention to adult responses to trauma and there has been no examination of these processes in children’s memory for traumatic events.

Another focus of memory research has been on the role of gender because of evidence of differential autobiographical memory patterns in men and women. Despite some mixed results in the literature (which can be attributed in many cases to methodological variations; [34], women tend to recall earlier, more detailed, and more emotionally rich autobiographical memories than men [35, 36]. It appears that in western settings at least, parents are more elaborative when reminiscing with daughters than sons [37]. Therefore, it is not surprising that young girls by the end of preschool tend to recount more detailed narratives than boys [14]. The role of gender may be relevant to cultural influences on childhood memory because of the differential roles played by gender across cultural settings. For example, one study of Asian, European, and Mauri adults found that earliest memories were reported latest by Asian participants, however this was due entirely to the late reporting of memories by Asian women [22]. This issue is particularly relevant in the context of Nelson and Fivush’s [21] social-cultural theory because it posits that the nature of autobiographical memories, along with one’s self-construct, is shaped by culturally-defined processes that determine how one perceives personal and societal histories. Consistent with this proposal, whereas in Western cultures it is common for children to be reared with the expectation to hold a coherent set of personal memories that define one’s past and describes one’s identity [38], this pattern does not hold in non-Western cultures [39].

This study seeks to extend this literature by exploring Acehnese children’s memories of the 2004 south-east Asian tsunami. Specifically, it aims to understand the nature of vantage point of trauma memories in children from a non-western culture, with particular focus on the role of gender. On December 26, 2004 a 9.3 underwater earthquake erupted, triggering a series of tsunamis that devastated more than 100km of Aceh’s coastline. The town of Meulaboh, where this study was conducted, suffered the highest casualties and damage to infrastructure. In Indonesia alone, over 126, 960 people, approximately 2% of Indonesia’s population, were confirmed dead by the World Health Organization [40]. Additionally, an estimated 35,000 Acehnese children were left homeless, orphaned or separated from their parents [41]. An interesting feature of this location is that it is strongly influenced by Sharia law, which involves different societal modelling for girls and boys. In brief, it has been noted that whereas girls are often encouraged to be acquiescent in their behaviour and in their expression of emotional responses, boys appeared to be afforded greater freedom to express themselves both verbally and behaviourally [42].

Given the age at which the tsunami occurred for many of the children in this study, we were also interested in understanding how young children may report awareness of the trauma when they do not directly recall being present at the event but rather reported hearing stories about it. Previous research has noted that the influence of media exposure on children’s posttraumatic responses [43, 44], suggesting that post-event information can have a marked impact on how children understand traumatic experience. Accordingly, we expected that although some children may not have encoded the trauma directly, the indirect exposure in the following years would have an impact on their psychological functioning, indexed by PTSD.

We studied children between the ages of seven and 13 years, five years after the tsunami. We hypothesised that analogous to adults, children who adopted an observer perspective would be associated with more severe PTSD compared with children who reported recalling the tsunami through their own eyes. On the basis that memories that have been reconstructed from other’s reminiscences of the tsunami would be understood from another’s perspective, we hypothesized that indirect memories would be more likely to be retrieved as observer memories rather direct memories. To explore the role of gender in this population, we analysed responses according to boys and girls, and also according to the age of the child at the time of the tsunami.

## Method

### Participants

Participants comprised 110 children (45 boys, 65 girls) between seven and 13 years of age (*M* = 10.43, *SD* = 1.38), living in Meulaboh. Table 1 provides a summary of the extent of trauma exposure and loss suffered by children, broken down by gender and age group (young and old). According to children’s reports, older children (aged ten to 13 years) sustained more losses and were exposed to greater threat during the tsunami. This is not surprising, given the younger age group (seven to nine year olds) would have been toddlers at the time of the tsunami and more likely to be carried by a parent or physically protected by an adult. Alternatively, they may have had limited awareness of the extent of the threat at the time and this may bias their recall.

**Table 1:** Trauma Exposure and Loss in Children by Gender and Age.

	Age 7–9 years		Age 10–13 years	
	Male	Female	Male	Female
Living in an orphanage	1	1	6	5
Experienced death of family or friend from tsunami	5	8	14	36
Trauma exposure during the tsunami				
Nearly drown	0	0	2	1
Sustained physical injury	0	1	2	4
Separated from family	3	2	22	17
Home destroyed	3	5	18	21

All children attended after-school activities organized by a local non-government organization (Centre Mulia Hati (CMH)), with which the research team was collaborating. Children’s parents rarely attended such activities, and therefore this study was unable to gather comparative data from parents.

All the children attending the after-school program on the day of testing agreed to participate. Ten children were omitted from the following analyses as they either reported no recollection of the tsunami or did not respond to the memory quality items.

### Time of Data Collection

Children were interviewed approximately five years after the tsunami. In the immediate aftermath of the tsunami the west coast of the archipelago was besieged with humanitarian aid organisations and media attention. Aceh remained the focus of attention for a year afterwards, with much community attention again intensifying at the disaster’s anniversary. Consequently, it is likely that children’s indirect exposure to the tsunami through media exposure and hearing conversations about the disaster also intensified at this time. It is noteworthy that qualitative assessments [45] conducted with the same children and a sample of adults (including teachers, parents and other adults in the community) revealed frequent conversation about the tsunami even five years afterwards. Children and adults reported ongoing fears of the tsunami happening again, and that many in the community would avoid the sea when it is raining.

### Measures

All questionnaires were translated into Bahasa Indonesian by an accredited translator and also verified for accuracy and comprehension by a bilingual local mental health worker, and subsequently through back-translation. The following measures were delivered as part of a larger assessment battery and therefore brevity was paramount.

The Children’s Revised Impact of Event Scale-13 (CRIES 13;[46]) is a 13-item measure of PTSD symptomatology, on which symptoms are rated on a four-point scale how frequently they have experienced each symptom in the last seven days (1 = *not at all*, 4 = o*ften*). The measure has demonstrated validity and reliability in varying cross-cultural populations [46–48], and has strong internal consistency for the intrusion, avoidance and arousal subscales (*α* = 0.70; *α* = 0.73; *α* = 0.60, respectively) and for the full scale (*α* = 0.80) [46]. Subsequent analyses have confirmed that many of the items on the arousal subscale load heavily on the intrusion subscale and that the omission of the subscale did not decrease the efficiency of the measure [46]. Given these findings, prevalence rates of probable PTSD were calculated using the intrusion and avoidance scales. A cut-off score of 17, which denotes very probable PTSD, has demonstrated maximum sensitivity and specificity [46, 48] and was applied in the present study. In terms of the current sample, the internal inconsistency in this Acehnese sample was moderate for the intrusion, avoidance and arousal subscales (*α* = 0.43; *α* = 0.36; *α* = 0.35, respectively) and for the full (*α* = 0.54) scales.

No established assessments of memory had been validated for this population and given the time constraints, we developed single-item questions. For these reasons, children were primed to think about their memories of the event with the following instructions that were based on previous studies exploring vantage point of trauma memories [29, 30].

Some children can remember the tsunami. They might have images of what they saw at the time. Other children are unable to personally remember the tsunami but they know what happened from what others have told them about the tsunami.

Children were then asked, *do you personally remember the tsunami*? They indicated their response by circling *yes* or *no*.

Children were asked to think back to their memory of the tsunami and indicate whether they could *see it through your own eyes*, *so you cannot see yourself* or *see it as though you are outside your body*, *like a spectator or onlooker*, *so you can see yourself and what you are doing*. Children were informed that they could indicate experiencing both perspectives. Following prior coding systems of vantage memories responses were scored on a 3-point scale (1 = *through my own eyes*, 2 = *both through my own eyes and also as an onlooker*, 3 = *as an onlooker*) [49].

Children were also asked to rate on a 10-point scale (1 = *not at all*, 10 = *very often*) how often they heard stories about the tsunami.

### Procedure

The study was approved by the University of New South Wales Human Research Ethics Committee (# HREC 11021), and approved the following informed consent procedure. All children participating in an after-school program were invited to complete the survey. Parents or caregivers gave verbal permission for children to participate in both the after-school program and the current study. Written consent was not obtained from parents or caregivers because of high levels of illiteracy. Written documentation was noted of the verbal consent provided by the parents or caregivers. All children were instructed that participation was voluntary and they could decline to complete the survey at any time; no students declined or failed to complete the survey, although 10 children did not complete the memory items. Questionnaires were completed in a group format, led by trained local health workers known to the children. To assist those with limited literacy, items were read aloud to children and with Acehnese translation when children had difficulty comprehending Indonesian words. The questionnaires were completed in November 2009, approximately five years after the tsunami.

## Results

### Levels of PTSD

Fifty-three children (48%) endorsed the required intrusion and avoidance items on the CRIES-13 to indicate a high probability of receiving a diagnosis of PTSD. There was no significant difference between boys and girls. Similarly, analysis of probable PTSD by vantage point did not detect any significant differences.

### Reports of Reminiscing of the Tsunami

When asked to rate on a 10-point scale, both boys (*M* = 6.20, *SD* = 3.00) and girls (*M* = 6.06, *SD* = 2.95) reported frequently hearing stories about the tsunami, *t* (108) = 0.20, *p* = .81.

### Memory Reports

Two children stated that although they knew about the tsunami, they did not know what happened on the day of the tsunami; that is, they had no personal memories or knowledge of the day. Table 2 presents the number of children who reported memories and vantage points of their memory. Of those who responded, 33 children (33%) indicated an indirect memory of the tsunami (i.e. they knew what occurred on that day without personally recalling it), while 67% (*n* = 67) indicated that they could directly recall the event. Not surprisingly, marginally fewer children who were four years or younger at the time of the tsunami (48%) reported direct memories of the event than those who were at least five years old at the time (68%), (χ^2^ = 3.00, p = .08).

**Table 2:** Number of Children Reporting Direct Memories and Vantage Point.

Vantage Point	Direct Memory (%)	Indirect Memory (%)
Own Perspective	25 (96)	1 (4)
Both Perspectives	18 (90	2 (10)
Onlooker Perspective	24 (44)	0 (56)
Total	67 (67)	33 (33)

More children (97%; *n* = 30) who reported an indirect memory of the tsunami said they recalled the tsunami from an onlooker’s perspective to some extent (either fully or partially from an onlooker’s perspective) than those who recalled the event directly (63%; *n* = 42), (χ^2^ = 13.51, p < .001).

### Role of Gender

Table 3 presents the memory reports according to gender. Significantly more girls (81%; *n* = 51) directly recalled the tsunami than boys (40%; *n* = 18), while boys were more likely to rely on stories from others to reconstruct a memory of the tsunami (χ^2^ = 19.08, p < .0001). Boys were significantly more likely to adopt an observer perspective to some extent when recalling the tsunami compared to girls (χ^2^ = 15.45, p < .0001).

**Table 3:** Number of Children Reporting Direct Memories and Vantage Point According to Gender.

	Girls^a^ (%)	Boys^b^ (%)
	**Direct Memory**	
Direct Memory	51 (81)	18 (40)
Indirect Memory	12 (19)	27 (60)
	**Vantage Point**	
Own Perspective	23 (41)	3 (7)
Both Perspective	10 (18)	11 (24)
Onlookers Perspectives	23 (41)	31 (69)

^a^ N = 55,

^b^ N = 45

### Memory and Psychological Adjustment

To determine the relationship between memory responses and psychological adjustment, separate linear regressions were conducted to predict CRIES-13 and depression total scores respectively. Since there were different memory patterns in boys and girls, the relationship between memory characteristics and PTSD and depression severity was indexed separately for each gender. These analyses were only performed on children who reported direct recall of the tsunami because of the collinearity between indirect awareness of the disaster and observer vantage perspective. Separate multiple linear regressions were conducted for girls and boys that entered age at Step 1 (to account for developmental factor), the total number of deaths the child experienced from the tsunami at Step 2 (to account for the impact of loss on posttraumatic stress), and vantage point at Step 3. Tables 4 and 5 present the summary models of the PTSD regressions for boys and girls, respectively. The overall model was significant for boys (*F* (3, 13) = 8.81, *p* = .002), with the extent to which boys engaged in an observer perspective of the memory accounted for 43% of the variance of PTSD severity scores; specifically, an observer perspective was associated with lower PTSD scores in boys. The model was not significant for girls. Tables 6 and 7 present the summary models of the depression regressions for each gender. The models were not significant for either boys or girls in predicting depression.

**Table 4:** Linear Regression Analysis of Memory Characteristics and PTSD in Boys.

	*B*	SEB	*β*	*t*	*p*
**Step 1: Direct memory**	2.63	2.11	.18	1.25	.22
**Step 2: Age**	.25	.70	.05	.35	.73
**Step 3: Total deaths**	2.32	1.15	.27	-2.02	.05
**Step 4: Vantage point**	-6.99	1.92	-.51	-3.64	.001

*Note*. Step 1 R^2^ = .00, Δ R^2^ = .00. Step 2 R^2^ = .00, Δ R^2^ = .00. Step 3 R^2^ = .08, Δ R^2^ = .08. Step 4 R^2^ = .31, Δ R^2^ = .24.

**Table 5:** Linear Regression Analysis of Memory Characteristics and PTSD in Girls.

	*B*	SEB	*β*	*t*	*P*
**Step 1: Direct memory**	.82	2.48	.05	.33	.74
**Step 2: Age**	-.04	.71	.00	-.05	.96
**Step 3: Total deaths**	1.74	1.23	.21	1.42	.16
**Step 4: Vantage point**	-6.89	1.29	-.09	-.57	.57

*Note*. Step 1 R^2^ = .00, Δ R^2^ = .00. Step 2 R^2^ = .00, Δ R^2^ = .00. Step 3 R^2^ = .05, Δ R^2^ = .05. Step 4 R^2^ = .06, Δ R^2^ = .01.

**Table 6:** Linear Regression Analysis of Memory Characteristics and Depression in Boys.

	*B*	SEB	*β*	*t*	*P*
**Step 1: Direct memory**	-2.22	1.11	-.33	-2.00	.06
**Step 2: Age**	.21	.37	.09	.56	.58
**Step 3: Total deaths**	-.52	.60	-.13	-.86	.39
**Step 4: Vantage point**	-.13	.99	-.20	-.13	.99

*Note*. Step 1 R^2^ = .10, Δ R^2^ = .10. Step 2 R^2^ = .11, Δ R^2^ = .01. Step 3 R^2^ = .13, Δ R^2^ = .02. Step 4 R^2^ = .13, Δ R^2^ = .00.

**Table 7:** Linear Regression Analysis of Memory Characteristics and Depression in Girls.

	*B*	SEB	*β*	*t*	*p*
**Step 1: Direct memory**	-.05	1.49	.00	-.04	.97
**Step 2: Age**	.97	.40	.36	2.43	.02
**Step 3: Total deaths**	.05	.75	.01	.07	.94
**Step 4: Vantage point**	-.85	.72	-.18	-1.19	.24

*Note*. Step 1 R^2^ = .00, Δ R^2^ = .00. Step 2 R^2^ = .13, Δ R^2^ = .13. Step 3 R^2^ = .13, Δ R^2^ = .00. Step 4 R^2^ = .16, Δ R^2^ = .03.

## Discussion

Although most Acehnese children could personally recall the tsunami, approximately one third of the sample reported that their memories were composed from hearing stories about it from others. We premise our discussion with recognition that we did not corroborate whether children’s memory were direct or indirect. As it was not possible to obtain caregivers’ independent reports of whether their children were directly exposed to the events occurring during the tsunami, we emphasize that our comments are somewhat speculative. However, this finding underscores the reconstructive nature of trauma memories, and accords with evidence of people creating narratives of their traumatic experiences as time progresses [50](45). Although not well documented, this observation highlights that children who undergo trauma at an early age may be susceptible to the reconstruction of the traumatic experience because (a) they may not have the cognitive resources to adequately consolidate the memory when it is initially encoded, and (b) they may be more susceptible to the contextual influences of other retrospective reports of the trauma, due to a limited knowledge base [7, 51]. In the time elapsed between the tsunami and the current study, indirect exposure to stories about the tsunami was ongoing [52]. According to reports from focus groups and discussions with community leaders, this indirect exposure came from family and community members and through the presence of media and humanitarian aid organisations (see [45]. Indirect exposure likely intensified when traumatic reminders (e.g. subsequent earthquakes and poor weather conditions) provoked anxiety in adults. This continuous exposure to stories about the day may also explain the preservation of these memories in many children, despite it being 5 years since the tsunami.

Most of these children reported viewing their memories from an observer’s perspective. Moreover, more children who reported reconstructing the memory from second-hand reports adopted the observer perspective than those who had first-hand memories. It is interesting to consider this finding in the context of Nigro and Neisser’s [53] initial definition of observer and field perspective; whereas first-person perspectives were conceptualized as images of the experience where “the scene appears from one’s own position…from roughly the field of view that was available during the original situation”, observer perspectives were defined as where “one seems to have the position of an onlooker or observer, looking at the situation from an external vantage point” (pp. 467–468). It appears that the children who heard about the tsunami from others understandably recalled the event more from another’s perspective because their mental representations of the experience were based on other’s perceptions.

There were significant gender differences in the reconstruction of the trauma memory. Girls were 15 times more likely than boys to directly recall the tsunami. Subsequently, boys were significantly more likely to adopt an observer vantage memory than girls were. Since the tsunami was so widespread and devastating, it is highly unlikely that boys and girls were exposed to different experiences that day and accordingly encoded the event differentially. A more parsimonious explanation is that post-tsunami elaboration of the event in the days and months afterwards may have differentially influenced how the girls and boys reconstructed what occurred. Parental elaboration of events has been found to influence memory recall in children as young as two years old, as well as the level of detail in their narratives [34]. Qualitative study of Acehnese children indicates that whereas girls are encouraged to suppress their emotions, boys are permitted to engage in more emotional expression [54]. This view appears to be reinforced by Sharia Law in operation in Aceh in recent years, which strictly regulates the traditional adoption of gender-appropriate roles for girls and boys. It is possible that girls were not encouraged to talk about the tsunami; in contrast, boys may have been afforded greater discussion and elaboration of the event, which facilitated adoption of an onlooker’s perspective. It is also possible that observer vantage may be associated with a form of avoidance, and it is possible that boys adopted this style more than girls in the period after the tsunami. It is not possible to determine the reasons for this finding but it underscores the critical role of gender in how boys and girls in Aceh reconstruct and manage trauma memories.

Contrary to our hypothesis, boys who adopted an observer’s perspective were more likely to manifest lower PTSD severity. This finding does accord with studies indicating that an observer perspective is associated with reduced emotional intensity [28, 31, 55]. Several possibilities exist to explain this pattern. First, an observer vantage point can be adopted as a form of cognitive avoidance [31, 53]. In one sample, higher avoidance scores of trauma survivors was found in those with an observer perspective [29]. Accordingly, it is possible that avoidance is motivating an observer perspective, which reduces distress. This explanation seems unlikely, however, because the overall PTSD severity was lower in the boys with an observer perspective and so there would be little motivation to avoid. Another more parsimonious explanation is that boys with an observer perspective had less severe PTSD because their perspective was more likely to have been constructed on the basis of second-hand reports, and so their mental representations of the tsunami may not have caused them the level of traumatic distress that affected others. The reason this pattern was not observed in girls is not entirely clear but it may be attributed to the impact of the greater preponderance of observer perspectives held by boys in the sample. Interestingly, this pattern existed for PTSD rather than depression, with significant models not being observed for either boys or girls in predicting depressive levels.

In terms of limitations of this study, it is noted that it is not possible to determine what events were encoded or the extent to which the children’s memories were accurate or were encoded directly or indirectly. Closer qualitative study of the memory reports would have been informative to determine the content, structure, vividness, and response to the memories that children had. Relatedly, we note that questioning young children about nuanced issues concerning vantage point and direct or indirect memories is inherently difficult, and the accuracy with which young children can achieve such introspective judgements has not been established. Adding to this, children were asked to recall the event 5 years later. Although natural forgetting is likely to have occurred during this time, children remained somewhat exposed to stories about the tsunami through media exposure, particularly at anniversaries, and through community discussion. Future study should also index the extent to which reconstructed memories of events learnt from others are experienced as intrusions, and play a role in ongoing distress. To achieve this, it would be instrumental to conduct longitudinal study of traumatized children and to assess their vantage point in relation to ongoing adjustment. Relatedly, the items used to index children’s memory of the event and vantage point was developed for the purpose of this study and not validated. Unfortunately, no established measures have been validated in this population and longer questionnaires could not be used due to time constraints. Secondly, this study was not afforded a comparison group, which would have allowed a more comparative commentary of the role of culture in the reconstruction of trauma memories in children.

Finally, these findings would have been more robust if we had caregiver reports about the extent and nature of discussions about the tsunami, and about the experiences of the children. This would have allowed for better validation of children’s exposure to stories about the tsunami and manifestation of PTSD symptoms. These limitations notwithstanding, the current findings highlight that at least in boys, observer perspectives of trauma are associated with less severe stress reactions to the traumatic experience. There is a need to further understand how across different cultural settings children recall traumatic experiences, how these can be shaped by environmental influences, and how memories may be differentially constructed in boys and girls.

## Supporting Information

S1 Dataset Participant characteristics and memory responses. (SAV)
